# Medicine shortages and challenges with the procurement process among public sector hospitals in South Africa; findings and implications

**DOI:** 10.1186/s12913-020-05080-1

**Published:** 2020-03-19

**Authors:** Cynthia Modisakeng, Moliehi Matlala, Brian Godman, Johanna Catharina Meyer

**Affiliations:** 1 Department of Pharmacy, Dr George Mukhari Academic Hospital, Private Bag, Pretoria, South Africa; 2 Division of Public Health Pharmacy and Management, School of Pharmacy, Sefako Makgatho Health Sciences University, Ga-Rankuwa, Pretoria, South Africa; 3 Division of Clinical Pharmacology, Karolinska Institute, Stockholm, Sweden; 4 Strathclyde Institute of Pharmacy and Biomedical Sciences, Glasgow, UK

**Keywords:** Procurement, Essential medicines, Shortages, Public sector hospitals, South Africa

## Abstract

**Background:**

Medicine shortages are a complex global challenge affecting all countries. This includes South Africa where ongoing medicine shortages are a concern among public sector hospitals as South Africa strives for universal access to healthcare. The objectives of this research were to highlight challenges in the current pharmaceutical procurement process for public sector hospitals. Subsequently, suggest potential ways forward based on the findings as the authorities in South Africa seek to improve the procurement process.

**Method:**

Qualitative in-depth interviews were conducted with 10 pharmacy managers in public sector hospitals in the Gauteng Province, South Africa. A thematic content analysis was performed, with transcripts coded by two of the authors. Coding was discussed until consensus was reached. Categories were developed and grouped into themes.

**Results:**

The ‘Procurement process’ emerged from the data as the overarching theme, rooted in three main themes: (i) The buy-out process that was used to procure medicines from suppliers other than the contracted ones; (ii) Suppliers not performing thereby contributing to medicine shortages in the hospitals; and (iii) Challenges such as the inaccuracy of the electronic inventory management system used in the hospitals.

**Conclusions:**

Effective management of contracts of suppliers by the Provincial Department of Health is crucial to ensure accessibility and availability of essential medicines to all citizens of South Africa. Ongoing monitoring and support for the future use of computerised inventory management systems is important to reduce medicine shortages, and this is being followed up.

## Background

Over the years, the shortage of medicines has been a challenge to the effective delivery of quality healthcare services worldwide including South Africa [1–10], with shortages of even essential medicines becoming a global problem irrespective of the economic status of countries [11–13]. More than half of the world’s population do not have access to essential services [14], exacerbated by medicine shortages. The origins of medicine shortages are complicated, can differ among regions and countries, and consequently potentially require different strategies to address them [5, 6, 15]. In the United States (US), the total number of new and ongoing shortages exceeded 450 in 2012 [12]. Overall in the US, as many as 99% of pharmacists in a study published in 2014 reported experiencing at least one shortage during the previous 12 months, 64% reported that their facility had completely run out of at least one injectable oncology drug during the past 12 months and 25% reported that one or more safety events had occurred at their facility as a result of shortages of medicines [16]. In Europe, a recent survey among hospital pharmacists showed there had been a significant increase in medicine shortages across Europe with 91.8% hospital pharmacists in 2018 experiencing shortages compared to 86.2% in 2014, with 35% experiencing them on a daily basis and 38% on a weekly basis [17], which is a concern. There are also considerable shortages in other continents and countries including other low and middle income countries (LMICs) across South America and wider [5, 6, 8, 9].

Some of the contributing factors to the shortage of medicines include manufacturing and quality problems, production delays, lack of manufacturing capacity, shortage of raw materials, political instability, and profitability issues [5, 6, 8, 9, 18]. Other factors include long lead times, delays in awarding tenders, absence of national contracts on the regional code lists of medicines, the failure of suppliers to meet demand, and the failure to pay suppliers [2, 19, 20].

The continuous and adequate supply of medicines is a key element in managing diseases especially chronic diseases [21], with the availability of essential medicines dependent on efficient supply chain systems among other factors [22]. Good systems are the ecosystem of organizations, with people, technologies, and activities brought together to ensure good delivery of medicines from manufacture right through to the end user, the patient, at affordable and acceptable prices [2, 6]. The key being to identify and rectify problems as early as possible. Regulatory issues contribute to the shortage of medicines especially if companies are unable to meet Good Manufacturing Practice and have their facilities closed down following an inspection of their facilities [23]. Early mandatory notification by manufacturers of any discontinuation or disruption in the manufacturing of a drug that could lead to a shortage is necessary in the management of medicine shortages are welcomed, with such systems in place in a number of European and other countries [5, 6, 24]. The Food and Drug Administration in the US adopted this particular strategy, which has resulted in quicker and more effective responses to medicine shortages [24].

Pharmacists, as the custodians of medicines, do play a critical role in the management of pharmaceuticals to help ensure the availability of medicines to patients in the public sector [25]. The pharmacy department especially in hospitals can also better manage the shortage of medicines by ensuring that in its organization, there are well defined management strategies. Infrastructures such as drug shortage teams, resource allocation committees, and processes for approving alternative therapies are important considerations in hospitals [26–28]. It is also important that healthcare institutions themselves conduct medicine shortage impact analysis in their institutions in order to understand the seriousness of current shortages as well as resources that are potentially available to contain the situation where possible [24]. Potential activities can include seeking ways to address the issue of, penalties not imposed on suppliers that failed to deliver within lead times, as well as addressing irregular ordering patterns by facilities from the depots [29]. This is an issue especially among sub-Saharan African countries including South Africa [10, 15, 22, 23]. The South African Healthcare system comprises both the public and private sectors. The public sector is run and managed by the government, rendering its services through public hospitals categorised by their levels of care as central, tertiary, district, and regional as well as clinics. This sector provides healthcare services to 82.5–84% of the population while only 17.5% of the population is serviced by the private sector [22, 30]. Healthcare services in the public sector are financed through general tax revenues while healthcare services in the private sector are largely financed by medical aid schemes with a small fraction financed through out of pocket payments [30]. The cost of medical schemes has kept the majority of the population from accessing the services in the private sector [31]; however, there is controversy regarding the extent of payments [32].

The selection of medicines in the public health sector in South Africa is performed by the National Essential Medicines List Committee (NEMLC) appointed by the Minister of Health [33]. Medicines selection is essentially based on evidence of efficacy, safety, quality and cost. However, considering limited financial resources, decision-making in terms of selection and procurement is informed by the anticipated disease burden, and subsequent budget impact analysis. With the future introduction of National Health Insurance, pharmacoeconomics evaluations and health technology assessment will be paramount to medicines selection [34].

The procurement of medicines and pharmaceutical supplies is undertaken through an open centralized tender system. The National Department of Health enters into contracts with the pharmaceutical suppliers on behalf of the provinces [33]. After the tenders have been awarded by the NDoH to the suppliers, the provincial depots perform the quantification and procurement of pharmaceuticals on behalf of the facilities in their Province. The depots are also responsible for warehousing and distribution of medicines to the facilities [29]. Although most medicines are delivered via this route, provinces are increasingly relying on direct delivery of medicines from contracted suppliers to health facilities, which shortens the supply chain and removes risks and costs associated with warehousing [35]. Buy-outs can however occur and this process is used by hospitals to purchase medicines appearing on the EML but not on tender. The buy-out process is also used when the contracted supplier does not perform as agreed [29], and is not a normal or standard method to purchase pharmaceuticals. Buy-outs typically occur as a result of late advertising and award of tenders resulting in considerable time and effort spent on this process [29]. Suppliers who are contracted by the NDoH through a tendering process are obligated to observe lead times, maintain good quality of products and supply complete orders. It is the duty of the provincial DoH to pay the supplier accounts within a 30 day period from the day of receipt of valid invoice as stipulated by the Public Finance Management Act (PFMA) no. 1 of 1999 [36].

The ongoing medicine shortages in public sector hospitals, especially in South Africa, highlight the need to explore this further [37, 38]. We are aware that there have been recent programs to help reduce potential medicine shortages in the public sector in South Africa [22] building on concerns in some provinces [25]. As a result, there is a need to gain current understanding of the experiences of pharmacy managers and drug controllers regarding medicine shortages in South Africa and potential ways to address this. Drug controllers, a post unique to the Gauteng province, and assistant managers occupy specialized posts in central and tertiary hospitals. Their role is to ensure effective and efficient medicines supply management services through formulary management in their institutions and provide expert advice and communication to management teams on matters relating to procurement, logistics management and use of medicines. They achieve this by making sure that there is no-over expenditure and that institutions adhere to Standard Treatment Guidelines (STGs) and EML [39]. Consequently, the purpose of this study was to highlight challenges in the pharmaceutical procurement process, reported to have contributed to medicine shortages in public hospitals in South Africa, to provide future direction.

## Methods

### Study design and population

This was a qualitative descriptive study with data collected through in-depth interviews. The target population included 32 people including 27 pharmacists who were pharmacy managers from the four levels of care (district, regional, tertiary and central) and 5 drug controllers/ assistant managers in the Gauteng Province. Gauteng province was selected for this initial study due to ease of access to key personnel in all four levels of care in public sector hospitals. Participants were then purposefully selected based on the number of years of experience in their positions. Request for permission to conduct the study in the public hospitals was sought from the Department of Health. After the Gauteng Ethics Committee granted permission for the study to be conducted, invitations were sent to the 27 hospitals in the Province 9 hospitals responded and agreed to participate in the study. Eight pharmacists were interviewed from 8 hospitals (one from each) and two pharmacists were interviewed in one hospital. None of the drug controllers were interviewed in the study. Three of the hospitals that had drug controllers did not respond to the invitation and two other drug controllers were not available to be interviewed at the time the study was conducted. Data saturation was reached regardless of the small sample size.

### Interview guide and interviews

An interview guide was developed specifically for this study in line with the study objectives (Supplementary file 1). The guide involved open ended questions that allowed probing about (i) the experiences of the pharmacy managers on the shortage of medicines in their hospitals; (ii) the impact of medicine shortages for pharmacy staff and patients; (iii) strategies used to manage medicines; and (iv) any changes to normal practices to address medicine shortages. Data were collected through in depth interviews aimed at gathering information on the medicines shortage phenomenon and facilitating fruitful discussions. All interviews were conducted by the first author in English, the language of communication in the workplace, between September and December 2017. Arrangements were made via the telephone for a suitable date for the interview at the participants’ workplace. Interviews took place in a private room with limited disturbance and were recorded using a digital voice recorder. Participants provided written informed consent for participation in the study and recording of the interviews.

### Analysis

The recorded interviews were transferred from the digital voice recorder to a computer and stored as Windows Media Audio files. Each interview was transcribed verbatim and saved as a Microsoft Word® document. The transcripts were then imported into NVivo 12® a qualitative data analysis software programme. Transcripts were then coded by the first author and re-coded by the second author. Continuous discussions took place between the two authors until a consensus was reached regarding the coding framework. A thematic analysis approach was used based on the hermeneutic theory where data were coded into ranks after a process of classifying codes and re-classifying sub-codes to ensure that exclusionary themes emerged [40, 41].

Verbatim quotations from participants, presented in italics text and enclosed in quotation marks, are used in the results section to illustrate the findings. Quotations were edited for punctuation to enhance readability and phrases or words removed from any quotation are replaced by three dots where pertinent. Pharmacists’ designations follow the quotations and are presented in square brackets.

### Ethics

Ethical clearance for the study was obtained from the Sefako Makgatho University Research Ethics Committee prior to the commencement of the study (SMUREC/H/44/2016: PG). Permission to conduct the study was granted by the National Department of Health (NDoH), the Gauteng Health Research and Ethics Committee and the Chief Executive Officers of the participating hospitals. Participants provided written informed consent.

## Results

### Sample characteristics

Ten pharmacy managers were interviewed, five from regional hospitals, one from a district hospital, two from tertiary hospitals and two from a central hospital. This sample was representative of the four levels of healthcare delivery by public sector hospitals in the Gauteng Province as well as generally among the public healthcare system in South Africa. Out of the ten participants, eight were females and two were males with age range of 36–61 years, and the average number of years of experience as pharmacy managers of 5 years.

### Overarching theme: procurement process

**Fig. 1:**
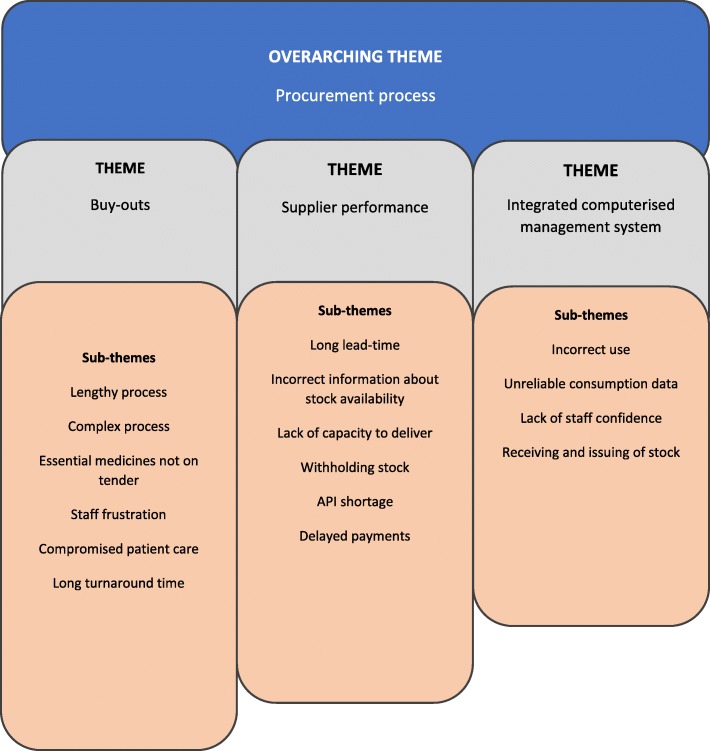
Themes on the procurement process derived from data

An overarching theme ‘Procurement process’ emerged from the data and is rooted in three main themes (i) Buy-outs; (ii) Supplier performance; (and (iii) Integrated computerised inventory management systems (RxSolution). The main themes are summarised in Fig. 1 and discussed in more detail below.

### Theme 1: buy-out process

Several participants raised concerns about the buy-out process in their hospitals. According to the participants, the buy-out process is currently very lengthy. In some instances, it did not ensure medicines availability at the time of need due to its complex nature. In fact, some participants felt that the buy-out process actually contributed to the problem of unavailability of medicines in their hospitals.

Overall, it was evident from the interviewees that the buy-out process puts considerable strain on hospital pharmacists, taking-up time that could have been invested in other important activities in the pharmacy including ensuring the rational use of medicines within the hospital.

Essential medicines that were not on tender contributed to a number of buy-outs that hospitals had to deal with. One of the participants suggested that the Central Medicines Depot should take over the buy-out of medicines listed on the EML on behalf of the hospitals to ease the burden of this process. The participants’ frustration is illustrated in the following narratives:

“*I don’t understand why the Depot is not doing buy-outs for vital and essential medicine items on the EML on behalf of the institutions, because the buy-out process is too much paper work. This process can easily take at least 4 weeks from the time the application goes in, to the time stock is received. Even though I am frustrated and irritated by this, I still need to do what needs to be done for patients to get their medication.*” (Regional hospital pharmacist 2).

“*Buy-outs are not the way out as such, because their turnaround time is too long. You have to wait for companies to give you quotations, you sign the documents and the CEO signs as well and you then send them to Medical Supplies Depot. You then wait for a committee that sits at the Depot that decides whether to approve your application or not, and the process takes time, six weeks at most. During that long period of waiting, the hospital would not have anything and patient care is compromised.*” (Regional hospital pharmacist 5).

### Theme 2: supplier performance

Several participants raised several concerns with the suppliers that they believed contributed to the medicine shortages. Participants reported that they waited for periods longer than 21 days before orders were received from the suppliers. In some instances, suppliers did not deliver full quantities of orders. Some of the participants reported to have received incorrect information from the suppliers about stock availability and this also resulted in medicine shortages in their hospitals. Participants pointed out that tenders were sometimes given to companies that did not have the capacity to deliver, which has important implications for patient care within their hospitals.

“*With the companies the lead time is 21 days, but you find that there are orders that would be outstanding for about three months because that particular company was awarded the contract and now it does not have stock. The company will claim that it has stock but when you place orders you find that they don’t have stock. So it is a serious problem.*” (Regional hospital pharmacist 3).

“*And the other thing was that the contracted companies were not able to supply the quantities they were contracted for. They could not meet the demand.*” (Regional hospital pharmacist 1).

Some participants alluded to the fact that suppliers were unable to deliver on orders placed because of the shortage of active pharmaceutical ingredients (APIs). As a result, stock-outs were experienced in their institutions. However, these shortages of APIs were not a South African phenomenon only.

“*The fundamental problem we had a year ago was the shortage of the APIs apparently worldwide, so it wasn’t just a problem for South Africa.*” (District hospital pharmacist 1)

Some participants stated non-payment of suppliers as a contributing factor to medicine shortages in their hospitals. They explained that suppliers who were not paid on time did not deliver the stock that was ordered to the hospitals. As a result, the availability of requested medicines was affected. It was evident from the participants that the suppliers did not typically communicate with the hospitals in advance on issues that have the potential of causing medicine shortages.

*“The suppliers were not being paid; and they stopped supplying because they were owed money. In fact, we are still trying to recover from that, even now. That has been the problem.”* (District hospital pharmacist 1)

Raising the same view, the regional pharmacy manager also highlighted the fact that they did not know what the problem was until they made a follow-up with the suppliers about outstanding orders.

*“Sometimes you would call the company to make a follow-up on the order and you would be told that your account has been put on hold because of issues of non-payment, and that does contribute to medicine shortages.”* (Regional hospital pharmacist 1)

The Public Finance Management Act (PFMA) no. 1 of 1999 stipulates that supplier accounts should be paid within a 30 day period from the day of receipt of a valid invoice [32]. Some participants stated non-payment of suppliers as a contributing factor to medicine shortages in their hospitals. They explained that suppliers who were not paid on time did not deliver stock ordered to hospitals. As a result, the availability of requested medicines was affected.

### Theme 3: integrated computerized inventory management system (RxSolution)

In 2014, the Gauteng Province implemented an electronic pharmaceutical management system, referred to as RxSolution, to manage the inventory in the public sector healthcare institutions. Four of the ten participants raised specific concerns with RxSolution. They complained that the pharmacy staff did not use the software appropriately making inventory management a difficult task. Stock was not received and issued correctly, and the consumption data produced by RxSolution was not reliable. This made it difficult to quantify orders.

Quantities ordered based on these unreliable consumption data by some hospitals subsequently led to medicine shortages because insufficient quantities that were ordered did not meet the needs of their patients. In some hospitals, staff also lacked confidence in the use of RxSolution. As a result, its use was minimal. Counting and verifying stock physically was also undertaken by storeroom staff due to the fact that the quantities produced by the software were not always reliable. However, one participant expressed a view that the use of the RxSolution system in their hospital helped with stock management. Information on soon to expire medicines was retrieved from the software. The short-dated stock report generated by the RxSolution system helped the hospital to redistribute stock to other hospitals that were in need of the stock and they were able to utilise these medicines before they expired. This helped in minimising waste in this hospital and improving medicines availability in other hospitals.

“*Yes, it (RxSolution) actually runs a report, and can tell you which items are expiring and when. That report helps us to know the short dated stock so that we can start redistributing it to other hospitals as well*” (Regional hospital pharmacist 4)

However, overall it became evident there were concerns generally with RxSolution.

“*We still need to work on the accuracy of the system. I am still not confident about it. The stock levels on the system are not the same as the stock levels on the shelves. We cannot rely on what is on the system therefore we always have to verify the quantities physically on the shelves.*” (Tertiary hospital pharmacist 2).

There were also concerns about updating the RxSolution system when issuing and receiving stock, and did not even keep physical records.

“*You think you’ve got stock because the bin card and the computer are telling you that, but actually there would be nothing on the shelves. People take stock and they don’t update the system and don not even record in the book, so the data on the system is inaccurate then it affects our ordering.*” (Central hospital pharmacist 2).

## Discussion

While the causes of medicines shortages are well known, we believe this is the first comprehensive study to explore pharmacists’ experiences with medicines shortages in South Africa. Our results have shown that the challenges experienced with medicines shortages did not discriminate against the different categories of the hospitals as they were similar across all levels of care. The main findings highlighted the challenges in the procurement process which included the non-payment of suppliers, poor supplier performance, a lengthy buy-out process, and a shortage of APIs. Our study also highlighted the challenges with the use of the integrated computerised inventory management system (RxSolution) used to manage pharmaceutical inventory in the hospitals: however this was not universal.

Whilst the PFMA no. 1 of 1999 in South Africa stipulates that suppliers should be paid within the 30 day period from receipt of a valid invoice, pharmacists reported that suppliers were not always paid on time and as a result, they did not supply the hospitals with their orders. Subsequently, hospitals waited for longer periods of time for their orders to be delivered. To compound the situation, as mentioned, suppliers did not typically inform hospitals in advance about possible medicine shortages due to accounts that were outstanding, with hospitals only getting to know about the problem of outstanding accounts when they follow-up on outstanding orders with the suppliers. These findings are similar to those of Muhia et al. in Kenya where the non-payment or late payment of suppliers was one of the key factors that affected the procurement of medicines [23]. The NDoH in South Africa in an attempt to prevent non-payment of supplier accounts posed penalties to Provinces that were found guilty of such conduct [15]. We will investigate this further in future studies to help reduce medicine shortages and their consequences. For instance, the Organization of Eastern Caribbean States (OECS) Pharmaceutical Procurement Service (PPS) was established in 1986 to manage the procurement process of member countries of the OECS to help address such issues. OECS/PPS procures pharmaceuticals on behalf of nine different countries through a tender process and deals with problems such as slow payments of supplier accounts. Hence, the OECS/PPS can provide guidance to countries, including South Africa, experiencing drug shortages due to non-payments by individual hospitals to suppliers [24]. In 2010, a task team appointed by the NDoH considered the challenges and opportunities for improvement of medicines procurement in South Africa’s public sector. One of its recommendations was the establishment of the Central Pharmaceutical Procurement Agency (CPPA). This agency would manage procurement contracts on behalf of the provinces, and provide oversight to the payment of suppliers of pharmaceutical and pharmaceutically related goods and services. The implementation of this recommendation by the NDoH could be a solution to non-payment of suppliers, thus reducing problems of out of stock medicines [29]. At the Provincial level, fully functional Medicine Procurement Units (MPUs) are a potential solution as they are responsible for contract management at the operational level, therefore increasing access and availability of medicines at lower costs [19, 22]. This development will be monitored in future studies.

As mentioned, participants reported that their hospitals waited for long periods of time before their orders were delivered, and in some instances, suppliers delivered quantities less than the order. In addition, pharmacists reported being given incorrect information about stock availability leading to medicine shortages similar to previous findings in South Africa [25]. This is also similar to findings in Zimbabwe, where delayed deliveries and non-deliveries of pharmaceuticals by suppliers resulted in situations of medicine shortages [42]. The late delivery of pharmaceuticals was also one of the key findings in the Auditor General′s report (2016), further confirming the findings of our study. Penalties were not imposed by some medical depots on suppliers for late delivery of pharmaceuticals, which needs to be followed up [19].

The management of supplier contracts after the tender award is crucial in ensuring supplier compliance and improving medicine availability. The strategy of proper contract management used by the Kenya Medical Supplies Authority (KEMSA) could be the solution to the problem of non-performance of suppliers in Gauteng [14]. The MPUs are an important feature of the reformed supply chains in South Africa and a full implementation of MPUs may help validate the orders from facilities, make follow up on orders easier, collate documents to create payment packs, manage supplier performance as well as facilitate facility and supplier interaction [22].

Buy-outs result from the factors such as non-performance of suppliers and certain essential medicines not on tender. The pharmacists reported being frustrated by the buy-out process as this process can be very long, involving considerable paper work and taking up considerable time that could be profitably spent elsewhere. As a result, the current long and complex nature of this process caused medicine shortages in the hospitals and patient care was compromised, similar to previous findings [29]. The NDoH should make it its goal to safeguard contract management and to improve medicines availability in public sector hospitals. Tenders awarded on time may reduce the number of buy-outs, ease the burden that comes with doing buy-outs at the institutional level and improve medicine availability. These are considerations for the future.

Shortage of APIs affects many manufacturers worldwide, causing medicine shortages and leading to concerns with patient management [5, 43, 44]. Shortage of APIs was cited by the pharmacists as a reason given by suppliers when they were unable to supply orders of certain medicines. The participating pharmacists understood that the shortage of APIs not only affected South Africa but many other countries around the world. The reason for this can be that there is a limited number of raw material suppliers globally with China and India being the major producers of APIs [12]. In the study by Heiskanen et al. [2], shortage of raw materials was a common reason for shortage of medicines in agreement with our findings. The shortage of medicines including a lack of APIs has been a challenge in many countries requiring Governments to collaborate and to coordinate efforts and to find global solutions [10]. In addition, pharmacists and others should also agree in advance potential alternatives that could be used to limit the impact.

In addition, at a summit in Accra in 2007, heads of states and governments of the African Union endorsed the Pharmaceutical Manufacturing Plan for Africa [45], with expansion of API manufacturing as one of the principal goals to improve access to affordable, safe and efficacious essential medicines in line with WHO suggestions for LMICs [46]. In 2017, South Africa planned to open a pilot plant for production of APIs however to date this has not materialised. Opening of this plant was going to be, an important first step towards reducing the potential for medicine shortages of essential medicines [47]. The latest manufacturing technology should help deal with technology barriers as African countries seek to produce more advanced APIs in the future similar to the situation in Brazil [48]. Another potential strategy to help address shortages of APIs would be the establishment of a global shortage notification system that would enable countries to better manage such shortages [12].

This study highlighted the challenges of inventory management with the use of the electronic inventory management programme (RxSolution) among public hospitals in South Africa. However, this was not helped by RxSolution being used inappropriately with some staff members not entering the stock that was received and issued into the system. As a result, unreliable consumption data were generated with subsequent order quantities generated by the system potentially being not enough and causing medicine shortages in these hospitals. In addition, some of the pharmacists reported that they were still not confident enough to use RxSolution, which was a concern. ChePa et al. (2017) highlighted there could be negative mind-sets and perceptions during the implementation of the electronic inventory management systems in hospitals making change hard to implement [49]. Consequently, effective change management strategies are crucial in the future to ensure a smooth transition to new technologies [49]. However, whilst the implementation of RxSolution in some hospitals in this study did not produce the desired results, its implementation at the Intermediate Hospital Oshikata in Namibia yielded positive results [50]. With improved accuracy of the stock cards, rational ordering of medicines and a reduction in emergency orders. As a result, providing direction for the future to help reduce medicine shortages among public sector hospitals in South Africa.

Overall based on our findings, we believe improved efforts should be made by the (NDoH) to manage supplier contracts during the pre-award and post-award process to avoid awarding tenders to companies that do not qualify to be suppliers. This builds on current initiatives to improve the supply chain and obtain low prices for medicines through tenders [22, 51]. The NDoH should make it a point that tenders are awarded for all medicines on the EML to minimise buy-outs. We also believe the NDoH should re-look at the buy-out processes and potentially remove this burden from hospitals through national discussions in order for hospital pharmacists to concentrate on other areas to improve patient care. Change management programmes should also be produced in advance to help ensure a smooth transition when Information Systems are being implemented, such as RxSolution, given the concerns identified. The culture of any hospital institution should be thoroughly understood before any change can be introduced to mitigate against resistance. In addition, there should be ongoing monitoring and support in the hospitals to make sure that the use of new systems such as RxSolution is maximised to help reduce medicine shortages in the future.

Purposive sampling was used in this study and therefore we acknowledge possible selection bias. This was however minimised by selecting participants based on the number of years’ of experience in managerial positions, to ensure that information rich participants were recruited. Interviewer bias was minimized through interviewer training prior to the first interview. Audio-recording of interviews further minimized interviewer bias, as it was not necessary for the interviewer to manually transcribe the responses during the interview. Furthermore, all data were re-coded by the second author, followed by review of codes and discussion between the two coders until agreement on the coding was reached.

We are aware of a number of limitations of this study. The sample size of pharmacy managers used as participants in the study was small and limited only to one of the nine provinces in South Africa. Because of the qualitative nature of this study, which involved a small sample size the aim is not to formulate a generalized hypothesis, but to extract the essence of the experience [40]. Consequently the findings of the study may not be generalized to all public sector hospitals in South Africa. Although the drug controllers, who often deal with logistics management, were part of the hospitals that took part in the study, they were not available for interviews. Consequently, we missed an opportunity of getting a perspective different from that of the pharmacy managers on the shortage of medicines. However despite these limitations, we believe our findings are robust and we would expect to see similar findings in other Provinces in South Africa.

## Conclusion

This study shows that the medicine shortages are still a reality that affect different levels of hospitals in South Africa. The determinants of medicines shortages included delayed payments to suppliers, non-performance of suppliers, and shortages of APIs. There were also concerns that RxSolution is not being used and understood in the majority of hospitals. Its limited use can cause medicine shortages in some instances. Ongoing monitoring and support for pharmacy staff is necessary for RxSolution to be used for proper and effective inventory management in the hospitals, and to improve medicines availability. There are ongoing programmes to address a number of these concerns.

## Supplementary information

**Additional file 1.** Interview guide.

## Data Availability

Depersonalised transcribed interviews are available from the corresponding author on reasonable request.

## Abbreviations

ASHP: American Society of Health System Pharmacists
CCMDD: Centralised Chronic Medicines Dispensing and Distribution
CEO: Chief Executive Officer
EML: Essential Medicine List
GAO: Government Accountability Office
GMP: Good Manufacturing Practice
MPU: Medicine Procurement Unit
NCS: National Cost Standards
NDOH: National Department of Health
NEMLC: National Essential Medicines List Committee
PHC: Primary Health Care
PTC: Pharmacy and Therapeutics Committee
SMUREC: Sefako Makgatho University Research Ethics Committee
STGs: Standard Treatment Guidelines
WHO: World Health Organization
