# Supplementary material for: Medicine shortages and challenges with the procurement process among public sector hospitals in South Africa; findings and implications
Source: BMC Health Serv Res. 2020 Mar 19;20:234. doi: 10.1186/s12913-020-05080-1 (PMC7082963; doi:10.1186/s12913-020-05080-1)
Supplement: Supplementary file 1 — Additional file 1. Interview guide. [file 12913_2020_5080_MOESM1_ESM.docx]

Appendix 5: Interview guide

Greet and introduce yourself to the participant. Welcome him/her to the interview and thank him/her for their willingness to meet with you and to participate in the study.

Elaborate on the aim of the study to the participant and obtain written consent from him/her to take part in the study before the interview commences.

Explain that the interview will be audio recorded as part of data collection, but assure participant that information provided will remain confidential.

Reassure the participant to speak freely and honestly so as to get a clear and relevant perspective for the study

**Pose the following questions to the participant:**

1. Can you please tell me in detail your experiences about medicines shortages in your institution in the past year, with specific reference to the percentage of medicines availability and classes of medicines that were affected?
2. What measures did you have in place in order to manage the shortage?
3. How frequent were the emergency orders placed in the past year?
4. Which of your normal practices did you have to change in order to deal with the shortage?
5. Which of these measures do you consider key to the management of medicines and why?
6. Were there any barriers in the management of medicines shortage that you have encountered? If so, how did you overcome them?
7. To what extent did the medicines shortage affect you and the pharmacy staff?

*Probes:*

1. *Did pharmacy staff experience any challenges when dealing with patients?*
2. *Was the working relationship between the pharmacy storeroom personnel and other
   health care professionals put under strain in anyway?*
3. What impact did medicines shortage have on the patients?

*Probes:*

1. *What financial implications did the medicines shortage have on the patients?*
2. *Was the patients’ level of confidence towards pharmacy affected?*
3. What recommendations will you make for the future should a similar situation arise?
4. Is there anything you would like to add?

I will be analysing the information that you and other participants gave me and will be writing a report on the findings of the study.

Thank the participant for his /her time.
